# Exercise-induced hypoalgesia (EIH) in response to different exercise intensities

**DOI:** 10.1007/s00421-022-04997-1

**Published:** 2022-07-09

**Authors:** Fabian Tomschi, Dennis Lieverkus, Thomas Hilberg

**Affiliations:** Department of Sports Medicine, University of Wuppertal, Wuppertal, Germany

**Keywords:** Pain, Physiology, Pain pressure threshold, Bicycle ergometer, Pain inhibition

## Abstract

**Purpose:**

Acute physical activity leads to exercise-induced hypoalgesia (EIH). The aim of this study was to investigate the effects of four different exercise intensities on EIH.

**Methods:**

25 male (age: 24.7 ± 3.0) subjects underwent four different exercise sessions on a bicycle ergometer for 30 min each at 60, 80, 100, and 110% of the individual anaerobic threshold on separate days in a randomized crossover design. Before, as well as 5- and 45-min post-exercise, pain sensitivity was measured employing pain pressure thresholds (PPT) at the elbow, knee, and ankle joints as well as the sternum and forehead. Besides, conditioned pain modulation (CPM) was conducted using thermal test- and conditioned stimuli before, 5-, and 45-min post-exercise.

**Results:**

A main time effect was observed regarding PPT at all landmarks except for the forehead with higher values observed 5 and 45 min post-exercise compared to the pre-values. Yet, no interaction effects occurred. CPM did not change in response to any of the intensities used.

**Conclusion:**

EIH occurs 5 and 45 min after exercise regardless of the intensity used at the joints and sternum which might be explained by local pain-inhibiting pathways and probably to a limited degree by central mechanisms, as no hypoalgesia was observed at the forehead and no changes in CPM occurred.

**Supplementary Information:**

The online version contains supplementary material available at 10.1007/s00421-022-04997-1.

## Introduction

In healthy, pain-free populations and also in patients suffering from chronic pain, physical activity can lead to exercise-induced hypoalgesia (EIH) (Vaegter and Jones 2020; Koltyn 2000). EIH describes an acute reduction in pain and pain sensitivity following exercise. It is thought to last for up to 30-min post-exercise and consent exists that this phenomenon occurs in healthy people following different types of exercise modalities (Rice et al. 2019). Yet, EIH is more variable in chronic pain patients where pain sensitivity may remain unchanged or even increase in response to exercise (Vaegter and Jones 2020). The physiological mechanisms underlying EIH are currently incompletely understood, but most likely include the endogenous opioid system (Koltyn 2000) as well as the endocannabinoid (Dietrich and McDaniel 2004) and serotonergic (Lima et al. 2017) system, the autonomic nervous system, and cerebral blood flow (Malfliet et al. 2018).

It was demonstrated that aerobic exercise, isometric exercise, and dynamic resistance exercise produce hypoalgesic effects (Naugle et al. 2012). However, these different exercise types seem to induce EIH in a different magnitude. A recent meta-analysis revealed that EIH is most robust and shows the largest effects when preceded by aerobic exercise. Dynamic resistance exercise induced small EIH effects, and this meta-analysis showed that isometric exercise did not induce any hypoalgesic effects (Wewege and Jones 2021).

Yet, when observing studies that evaluated the EIH effects in response to aerobic exercise, it becomes evident that results are mixed and show different effect magnitudes. One possible explanation suggested is that different exercise intensities lead to a different magnitude of hypoalgesic effects. It is generally believed that a dose–response relationship exists between the intensity of aerobic exercise and resulting EIH, and research suggests that higher intensities lead to a higher EIH response (Naugle et al. 2012; Micalos and Arendt-Nielsen 2016; Hoffman et al. 2004). However, despite the fact that many studies examined the phenomenon of EIH, there is limited understanding of the optimal intensity of aerobic exercise to induce hypoalgesia by pain-inhibitory systems (Naugle et al. 2012). A dose–response relationship using several different intensities was not evaluated in one study, but is rather based on the comparison between different studies using different methods. Hence, the aim of the present study is to compare the different effects of acute 30-min bicycle ergometer exercises on EIH employing four different intensities.

## Methods

### Ethics

The study and the used protocols were approved by the local ethics committee. These protocols are in line with the Declaration of Helsinki. Participants gave written informed consent to participate in the study.

### Study population and experimental design

**Fig. 1:**
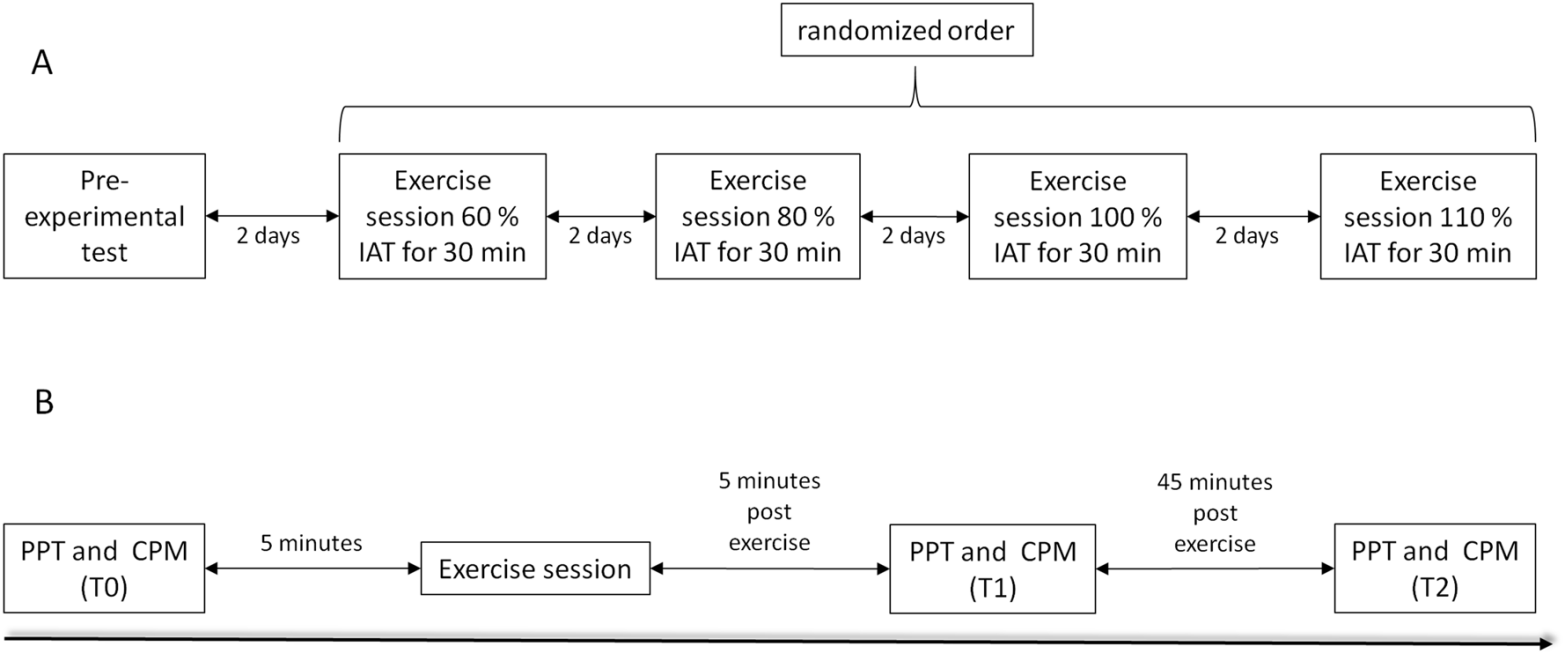
Flowchart of the study design using a randomized crossover design. **A** General overview of the exercise sessions; **B** overview of one exercise session including pressure pain threshold (PPT) and conditioned pain modulation (CPM) measurements. *IAT* individual anaerobic threshold

An initial sample size of 24 resulted from an a priori power analysis using G*power (Version 3.1.9.4) for a repeated measures ANOVA with four groups and three measurements. A power of (1 − *β*) = 0.90, an *α*-error probability of 0.05, a medium effect size of *f* = 0.33, and a correlation coefficient of 0.5 among repeated measurements were assumed. We further added a dropout rate of 25% resulting in the final sample size of at least 30 subjects. Hence, 31 male subjects were recruited for this study. Six subjects dropped out due to personal reasons and were not able to conduct all exercise sessions. 25 subjects successfully finished this study. Anthropometric data are presented in Table 1. Subjects were included if they were male, of the age of 18–30, and healthy. Subjects were excluded if they reported chronic or acute pain, suffered from any orthopaedic injuries, or were taking any analgetics. The study was designed as a randomized controlled crossover trial. A general overview of the study is presented in Fig. 1. In short, in the first visit, (pre-experimental test), subjects were checked for eligibility and subjects gave written consent to participate in this study. The pressure pain threshold (PPT) and the conditioned pain modulation (CPM) assessments were conducted to familiarize subjects with these assessments. Subsequently, trained medical staff conducted a medical examination including the assessment of the subjects’ individual anaerobic threshold (IAT) employing a cycle ergometry until exhaustion. In the following visits, subjects conducted four different interventions in a randomized crossover design. Every visit followed the same procedure: pain-related measurements (PPT and CPM) were conducted to assess subjects’ baseline values (T0). Then, the intervention exercise (60, 80, 100, or 110% of subjects’ IAT) was conducted. After the intervention, the pain-related measurements were repeated 5 (T1) and 45 min (T2) post-intervention.

**Table 1:** Anthropometric and performance data of participants (n = 25)

	Mean ± SD
Age (years)	24.7 ± 3.0
Height (cm)	181.7 ± 9.0
Weight (kg)	75.5 ± 7.8
Max. power (W)	291 ± 59
relative power (W/kg)	3.9 ± 1
Rel. VO_2_max (ml/kg/min)	50.3 ± 8.5
Max. heart rate (1/min)	188 ± 10
IAT (W)	203 ± 64
Lactate at IAT (mmol/l)	4.5 ± 1.3
Heart rate at IAT (1/min)	161 ± 12
Power at 60% IAT (W)	123 ± 38
Power at 80% IAT (W)	164 ± 51
Power at 100% IAT (W)	205 ± 63
Power at 110% IAT (W)	225 ± 70

Data presented as mean ± SD

*IAT* individual’s anaerobic threshold, *VO*_*2*_*max* maximal oxygen consumption, *W* watt power

### Pre-experimental test

In a pre-experimental test, a medical examination (12-lead resting electrocardiogram (ECG), stress ECG on a bicycle ergometer, blood pressure measurements, pulmonary function test, anthropometric measurements, anamneses, and a health-specific questionnaire (Physical Activity Readiness Questionnaire) was conducted by medical staff and a sports physician. PPT and CPM assessments were conducted to familiarize subjects with these procedures. The IAT was determined using an incremental stepwise protocol (start: 50 watts, increment: 50 watts every 3 min) on a bicycle ergometer (Excalibur Sport, Lode, Groningen, Netherlands) until exhaustion. Spirometric data were measured using the Vyntus CPX system (CareFusion, Hoechenberg, Germany). VO_2_max was recorded as the highest value of the measurement (Robergs et al. 2010). Lactate samples were collected at the earlobe at rest and every 3 min during the ergometer test as well as 1, 3, 5, and 10 -min post-exercise to calculate the IAT. Lactate samples were analysed using the EKF-Boisen S-Line Lab+ lactate analyser (EKF Diagnostics, Barleben, Germany). The rate of perceived exhaustion was recorded using the Borg scale at the end of each step and immediately after finishing the ergometer exercise. Heart rate (HR) was measured using an electrocardiogram (SEMA CS-200, SCHILLER Medizintechnik Gmbh, Feldkirchen, Germany) with the software program Cardiosoft (GE Healthcare, Solingen, Germany).

### Exercise sessions

Following the visit for the pre-experimental test, subjects visited the laboratory for another four times to conduct the exercise sessions with at least 48 h of pause between two test sessions to ensure sufficient regeneration time. Subjects were asked to refrain from vigorous physical activity 24 h before the respective exercise session. After 10 min of rest, the subjects’ PPT and CPM (T0) were assessed. Then, subjects conducted the respective exercise session, which lasted for 30 min. The intensities were set to 60, 80, 100, and 110% of the subjects’ IAT. The order of the separate sessions was randomly allocated. 5 (T1) and 45 min (T2), respectively, after having finished the exercise session, the PPT and CPM assessments were repeated, and subjects were asked to remain calm in a seated position. If a subject was not able to maintain the revolutions of 60 to 70 rounds per minute on the bicycle ergometer, the test was stopped, and the test duration was noted. The subsequent PPT and CPM assessments were conducted as usual, and data of these subjects were also included in the analyses.

### Measurement of PPT and CPM

All experimental measurements of PPT and CPM were conducted by the same investigator to avoid any inter-rater discrepancies. PPT measurements were conducted as described by Hilberg et al. using a handheld digital algometer (FPX 25 Compact Digital Algometer, Wagner Instruments, Greenwich, CT, USA) to detect subjects’ pain sensitivity. Via a 1 cm^2^ rubber tip pressure, pain was applied to different landmarks, i.e. the sternum, forehead and bilaterally to the elbow, knee, and ankle joints. Pressure was increased via an increase rate of 10 N (N) per second. Subjects were asked to report when the pressure stimulus first became painful. Peak applied force (N) was recorded. A cutoff value of 140 N was determined beforehand to prevent any tissue damage (Hilberg et al. 2011). The average value of three consecutive measurements (10 s of pause) was used for analysis and coefficient of variance values were subsequently calculated. If subjects did not report any pain under 140 N, a PPT value of 140 N was recorded (Krüger et al. 2016). For the assessment of EIH, PPT provides the most reliable results (Naugle et al. 2012).

The CPM test was conducted following the recommendations for CPM testing (Yarnitsky et al. 2015) with the aim of evaluating the descending pain inhibition pathway as described before (Krüger and Hilberg 2020). In short, the test procedure was divided into two test parts. In the first part (A), the individual stand-alone stimulus (test stimulus) was applied to the dominant forearm using a heat stimulus, which was applied for 30 s via a thermode via a 9 cm^2^ contact area (TSA-II; controlled via Thermal Sensory Analyzer 2001 TSA-II, Me-doc Ltd., Israel). The temperature of the test stimulus was calibrated on the day of the pre-experimental test. For this purpose, the thermode with an initial temperature of 32 °C was placed on the dominant forearm. After starting the test, the temperature increased by 1 °C per second. The heat to be applied was limited with a maximum heat value of 50 °C to avoid tissue damage. The test stimulus was to be classified by the subjects at a heat pain of 60 on a numerical rating scale (NRS) of 0–100. The software (TSA-II NeuroSensory Analyzer, version 6.1.19.4) indicated the applied temperature. This test was performed three times; the mean value was used for further analysis.

After a restitution pause of 1 min, the second part (B) of the procedure started. The cold pain stimulus (conditioning stimulus) was applied by immersing the non-dominant arm into a circulating cold-water bath (7°; basin B-18, 18 L; thermoregulator TE-10D; immersion cooler, RU-200; Techne, Staffordshire, UK) for one minute. After 30 s, the test stimulus was applied in parallel for 30 s. The application lasted 30 s from this point. The subject rated the severity of the test stimulus on a scale of 0–100 every 10 s. After 30 s, the thermode temperature decreased back to the baseline temperature of 32 °C. The CPM response was calculated by subtracting the NRS value from the heat pain from test part A from the NRS value from the heat pain from test part B, with negative values denoting pain inhibition.

### Statistics

Statistical analyses of the data were performed using the statistics software package SPSS 27 (IBM^©^, Armonk, NY, USA). The Shapiro–Wilk test was used to test the normal distribution with no need for further transformation. The Levene test was conducted and the homogeneity of variance was confirmed. To detect the effects of the four intervention protocols on PPT (sternum, forehead, elbows, knees, and ankles) and CPM, respectively, a two-way ANOVA with the factors ‘protocol’ (60%, 80%, 100%, 110%) and ‘time point’ (T0, T1, T2) was calculated to detect the effects of the different protocols on PPT and effects of the ‘protocol’ × ‘time point’ interaction. Effect sizes are presented as partial eta-squared (*η*^2^_partial_) with values of 0.01 representing a small, 0.06 a medium, and ≥ 0.14 a large effect, respectively (Cohen 1988). If differences were observed, Bonferroni post hoc analyses were conducted. Moreover, when main effects for the factor ‘time point’ were observed for PPT, subsequent LSD post hoc tests were employed for the respective landmark and intervention. Differences were considered significant with *p* ≤ 0.05, unless otherwise marked.

## Results

25 subjects successfully finished the study and conducted all exercise sessions. All subjects were able to finish the exercise sessions with 60 and 80% of the IAT, respectively. Five subjects did not finish 30 min of the exercise session consisting of 100% IAT. Therefore, the subjects finished this session after a total mean time of 27.6 (± 5.0) min. Besides, 16 subjects did not finish the 110% IAT session and the participants finished this session after a mean time of 22.5 (± 9.0) min. The time span between two sessions was 7.8 days (± 5.6) and ranged between 2 and 24 days.

**Fig. 2:**
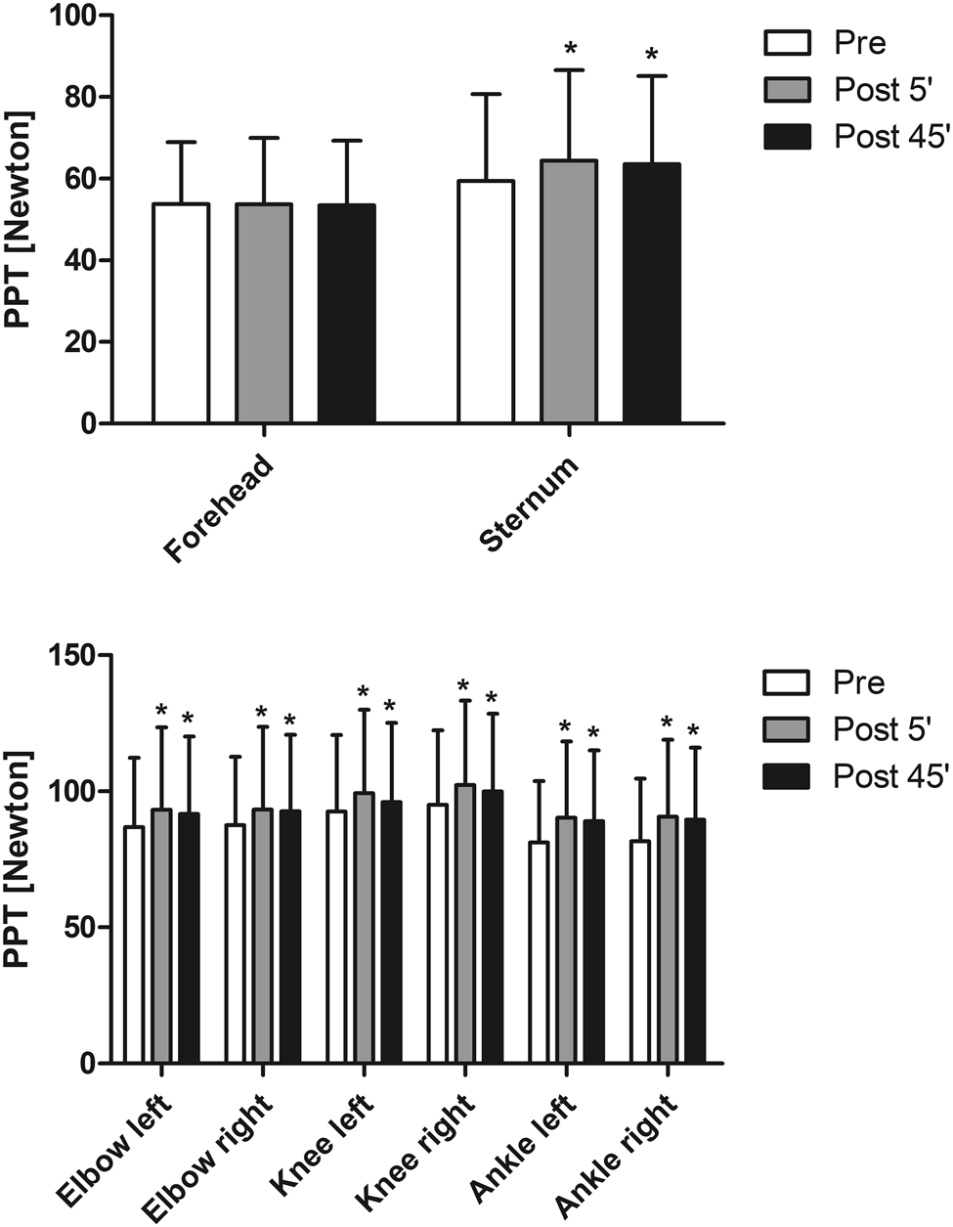
Means of pressure pain thresholds (PPT) in response to the all exercises presented as one mean value. A main effect was observed for the variable time point (*p* ≤ 0.05) for all landmarks, except the forehead. Data are expressed as means ± standard deviation of the four different exercises. *Indicates significant difference (*p* ≤ 0.05) to ‘pre’ of the respective landmark

**Fig. 3:**
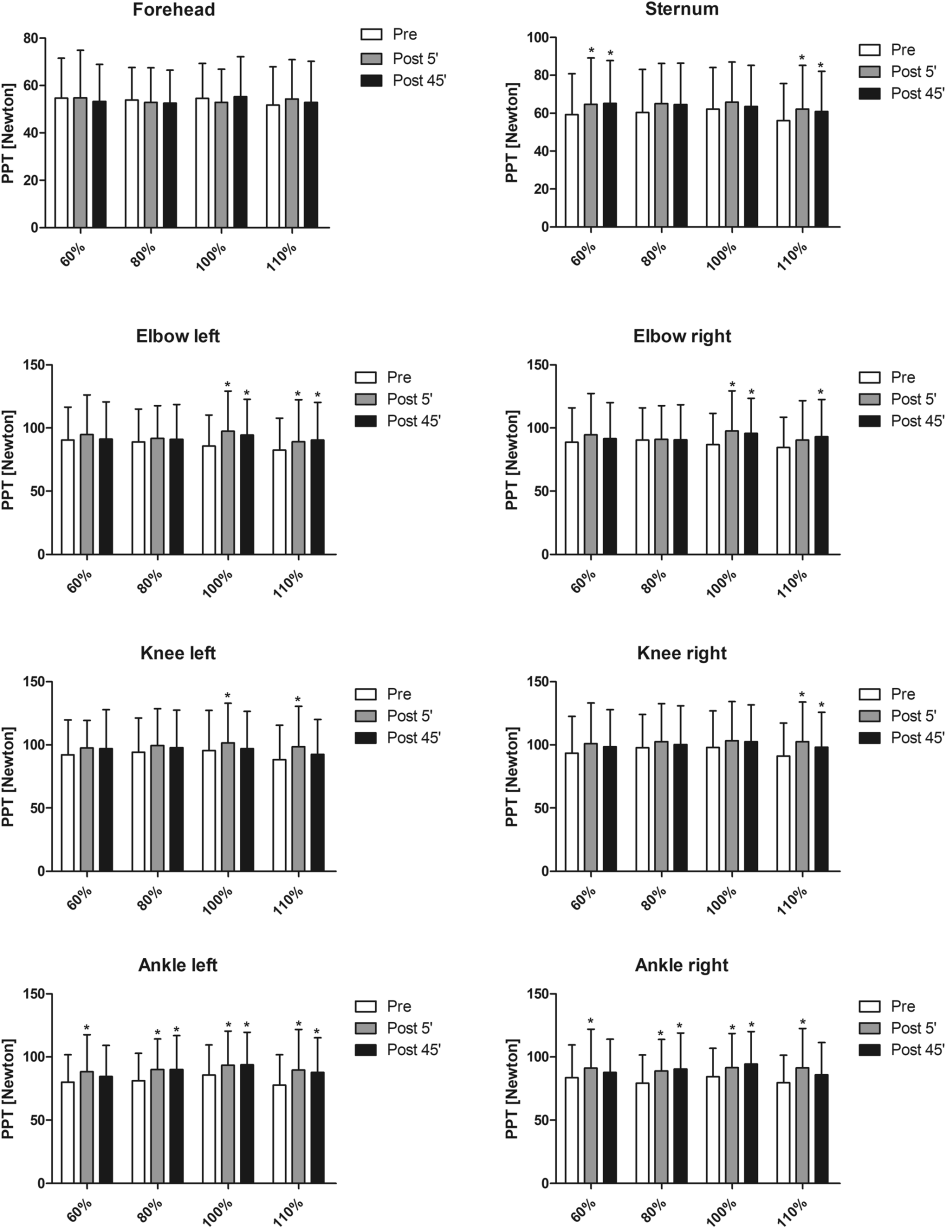
Pressure pain thresholds (PPT) at eight landmarks measured in response to the four different exercise intensities (*x*-axis: 60, 80, 100, and 110% of the individuals’ anaerobic threshold). No interaction effect (protocol × time point) was observed for any of landmarks. * indicates a significant difference compared to pre resulting from post hoc tests within the respective landmark and protocol (p ≤ 0.05). Data are expressed as means ± standard deviation

Statistical analyses employing a two-way ANOVA with ‘protocol’ and ‘time point’ as factors revealed no significant effect for the ‘protocol’ × ‘time point’ interaction of PPT of the forehead, sternum, left and right elbow, left and right knee, and left and right ankle. However, a significant main effect for the factor ‘time point’ was observed at the landmarks sternum, left and right elbow, left and right knee, and left and right ankle (see Table 2). Post hoc analyses revealed for each of these landmarks that PPT at T0 were significantly lower compared to T1 (*p* < 0.01) and T2 (*p* < 0.01), respectively. On the forehead, no main effect was observed for the variable ‘time point’. Means of PPT in response to all exercises are presented as one mean value in Fig. 2. Results of the subsequent post hoc tests employed for the respective landmarks and intensities revealing a main effect for the factor ‘time point’ are presented in Fig. 3 and Supplementary material 1. Coefficient of variance calculation revealed mean values ranging from 0.03 to 0.10 for the entire study. Further coefficient of variance values are provided in Supplementary material 2.

**Table 2:** Resulting *p* values and effect sizes (*η*^2^partial) of the two-way ANOVA (with the factors ‘protocol’ and ‘time point’) calculated for pressure pain thresholds (PPT) at the eight landmarks and conditioned pain modulation (CPM)

	Main effect ‘time point’	Main effect ‘protocol’	Interaction effect ‘time point × protocol’
	*p* value (*η*^2^_partial_)	*p* value (*η*^2^_partial_)	p value (*η*^2^_partial_)
PPT forehead	0.952 (0.002)	0.862 (0.010)	0.388 (0.042)
PPT Sternum	0.001 (0.258)	0.291 (0.050)	0.821 (0.020)
PPT elbow left	0.011 (0.205)	0.239 (0.057)	0.134 (0.065)
PPT elbow right	0.046 (0.133)	0.575 (0.027)	0.071 (0.084)
PPT knee left	< 0.001 (0.280)	0.278 (0.052)	0.717 (0.025)
PPT knee right	< 0.001 (0.303)	0.476 (0.034)	0.737 (0.024)
PPT ankle left	< 0.001 (0.462)	0.253 (0.055)	0.777 (0.022)
PPT ankle right	< 0.001 (0.367)	0.588 (0.026)	0.280 (0.050)
CPM	0.832 (0.008)	0.423 (0.038)	0.809 (0.020)

**Fig. 4:**
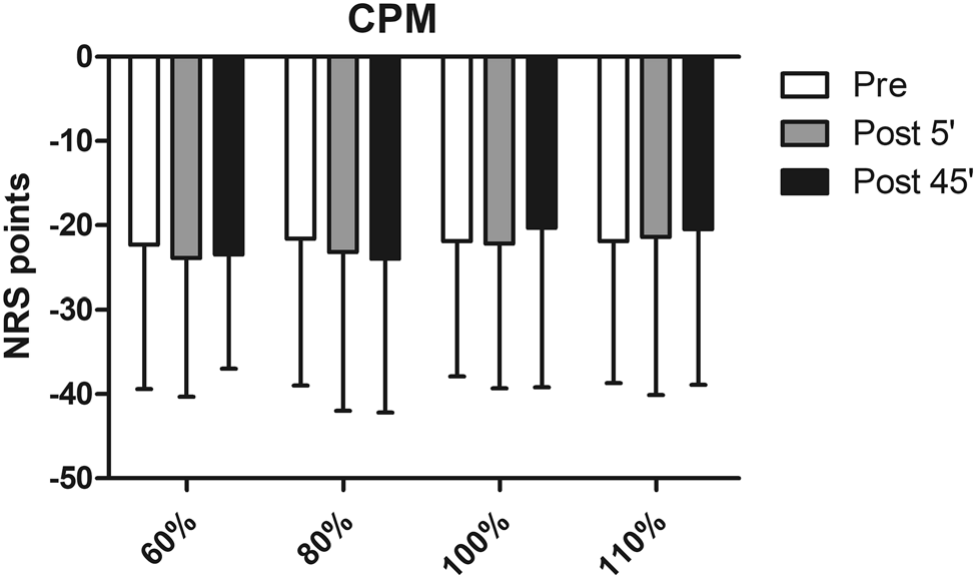
Results of the conditioned pain modulation (CPM) test following the four different exercise intensities (*x*-axis: 60, 80, 100, and 110% of the individuals’ anaerobic threshold). No statistically significant difference was observed. Data are expressed as means ± standard deviation. *NRS* numeric rating scale

Statistical analyses with protocol and time point as factors revealed no significant effect of the protocol × time point interaction of CPM. Further, no main effect was observed for the variable time point alone (Fig. 4 and Table 2).

## Discussion

This study’s primary objective was to evaluate the effects of four different aerobic exercise intensities on EIH measured using PPT. The results show a main effect over time indicating that EIH occurred at the landmarks of the joints and the sternum. Yet, no interaction effects were observed and, hence, a specific dose–response relationship cannot be established based on the results obtained. However, the results of the post-hoc calculations reveal that EIH seems to be more present in response to higher intensive exercise, i.e. 100 and 110% sessions, respectively (Fig. 3). Several studies investigated the effects of bicycle exercises on EIH using PPT measurements and employing certain intensities. The results of these studies predominantly show that the used exercises led to hypoalgesic effects on certain body sites after bicycle ergometer training. Hoffman et al. compared the effects of three exercise interventions on pain ratings of a 2-min pressure stimulus before and 5 min and 30 min after exercise. The interventions were performed on a treadmill and consisted of a 10-min exercise at 75% of maximal oxygen uptake (VO2max), a 30-min exercise at 50% VO2max, and a 30-min exercise at 75% VO_2_max. Pain ratings were significantly decreased 5-min post-exercise at 75% VO2max, while the other two interventions showed no effect. The authors concluded that there might be thresholds for intensity (> 50% VO_2_max) and duration (> 10 min) for aerobic exercise to induce acute hypoalgesia (Hoffman et al. 2004). Further, Naugle et al. showed that 20 min of vigorous exercise at 70% of HR reserve increased PPT, whereas PPT was unaltered after moderate exercise at 50% of HR reserve (Naugle et al. 2014). A study by Vaegter et al. also revealed that a high-intensity bicycle exercise at a calculated intensity of 75% VO_2_max led to a larger EIH response compared to a low-intensity exercise conducted at 50% VO_2_max (Vaegter et al. 2014) supporting the results presented herein with EIH effects observed primarily in the 100 and 110% sessions. Acute exercise most likely reduces pain sensation due to the release of analgetic endogenous opioid-related substances which are expressed centrally in the nervous system and, in addition, locally close to the contractile musculature attenuating nociceptive signalling (Micalos and Arendt-Nielsen 2016). This leads to the assumption that the magnitude of EIH is highest in body parts near the exercising musculature and weaker at remote body parts (Rice et al. 2019). This assumption is also observed in the present study with the highest EIH effects observed at the ankle joints (Fig. 3).

Some studies also demonstrate that acute bicycle exercise results in hyperalgesia, i.e. increase in pain sensitivity, following bicycle exercise. Krüger et al. demonstrated that an incremental bicycle ergometer session up to exhaustion resulted in hyperalgesia at the sternum and forehead. Neither hypo- nor hyperalgesic effects were observed at the elbow, knee, and ankle joints measured via PPT (Krüger et al. 2016). One might argue that the nature of the incremental bicycle session in which a high-intensity and anaerobic demand only occurs for the last 5–6 min is too short to induce hypoalgesic effects. Hyperagesic effects measured via PPT at local and remote musculature were also observed in the study by Micalos and Arendt-Nielsen after participants conducted a 30-min bicycle exercise at 30% of their VO_2_max. The authors state the theory that low-intensity exercise might increase the sensitivity of noxious stimuli by facilitating ascending afferent signalling (Micalos and Arendt-Nielsen 2016). However, studies also show that acute bicycle exercises might neither lead to hypo- nor hyperalgesia when measuring pain at the index finger after a 30-min steady-state cycling test at 75% of the VO_2_max (Monnier-Benoit and Groslambert 2006) supporting the above-mentioned assumption that hypoalgesia occurs more prominently at body parts close to the exercising muscles. The results of the present study reveal that all intensities induced hypoalgesia with a main effect observed at the joints and sternum. Compared to other studies that tried to examine the effects of exercise intensity on hypoalgesia, we chose the intensities ranging from 60% up to 110%. Hence, our lowest intensity is still higher compared to, for instance, the lowest intensity used in the above-mentioned study by Micalos and Arendt-Nielsen, who explored exercise-induced analgesia employing cycling exercises of 30 min at an intensity of 70 and 30% of peak oxygen uptake (VO_2_peak). They found that exercising at 70% of VO_2_peak attenuated pressure pain sensitivity locally at the rectus femoris compared to the hyperalgesic effects observed at 30% of VO_2_peak (Micalos and Arendt-Nielsen 2016). Higher-intensity exercise might influence the midbrain periaqueductal grey (PAG) and the rostral ventromedial medulla (RVM) network by modulating central pain transmission, which is associated with central endogenous release of inhibitory neurotransmitters in the central nervous system, such as GABA, leading to an inhibition of pain signalling (Micalos 2014; Micalos and Arendt-Nielsen 2016). This theory is supported by animal models showing that GABA increases in response to exercise in rats (Hill et al. 2010). Our lowest intensity exercise of 60% VO_2_max for 30 min might still be high enough to induce, at least to a certain degree, these proposed physiological reactions. Our results further indicate that the higher intensive protocols led to a broader EIH effect, as EIH is observed at more landmarks in the 100 and 110% sessions, respectively (Fig. 3).

The present study revealed that EIH occurred at the joints and the sternum. Yet, no change in exercise-induced analgesia occurred at the forehead. Our results show that EIH is higher, in terms of effect sizes, at landmarks near the musculature that is primarily engaged in the exercise supporting previous research (Vaegter et al. 2014). EIH effects may be less pronounced and may occur less consistently depending on the distance to the primary exercising body parts and musculature (Gomolka et al. 2019). Accordingly, the results of our study regarding hypoalgesia measured at the knee and ankle joints support this theory. Yet, a main time effect was also observed at the elbow joints and the sternum. One might argue that bicycle exercise also goes along with contractile activity in the arm and chest musculature. However, these muscle sites are by far less engaged in the total force production compared to the lower extremity musculature. Krüger et al. observed that an incremental bicycle exercise test up to exhaustion led to hyperalgesic effects measured by PPT at the sternum and forehead whilst no differences were observed at the same joint landmarks used in our present study (Krüger et al. 2016). Interestingly, our study revealed that neither hypo- nor hyperalgesic effects occur in response to any of the exercise intensities used at the forehead. The reasons for this observed phenomenon remain elusive; yet, it might be explained by the above-mentioned observation as the head is the most remote body part from the exercising musculature. To the best of our knowledge, only very little research was conducted using the forehead as landmark for hypoalgesic testing and more research is needed to further elaborate this observation.

Decreased pain sensitivity was observed 5-min post-exercise lasting up to 45-min post-exercise. It is generally believed that EIH effects employing PPT last up to 30 min (Rice et al. 2019). Our results indicate that the EIH time span might be even longer than generally believed, which is in contrast to previous research showing that EIH is only observed 5-min post, but not 30-min post-exercise (Hoffman et al. 2004). In another study, EIH was observed 5-min post, but not 15-min post-exercise (Micalos and Arendt-Nielsen 2016). Gomolka et al. conducted a study to evaluate the test–retest reliability of EIH using two 15-min exercise sessions. PPT were measured before, immediately after, and 15 min after at, among others, the leg. Results show that EIH occurred 5-min post in both sessions. Interestingly, 15-min post-exercise, EIH was only observable in one of the two tests (Gomolka et al. 2019). Yet, these studies mentioned are only comparable to a limited extent with each other and with the study presented herein as they differed in the methodological approach chosen. Differences in the exercise type (i.e. treadmill running, bicycle riding) as well as duration (i.e. 30, 15 min) and intensity (i.e. 75% VO_2_max, 70% VO_2_peak, or heart rate of 85.9% of age-related maximum) need to be considered. Further, experimental pain measurements employed were different with different methods (PPT or NRS response to a 2-min pressure pain stimulus to the finger) and body sites (finger, rectus femoris, or biceps femoris) used making it difficult to compare the results (Hoffman et al. 2004; Gomolka et al. 2019; Micalos and Arendt-Nielsen 2016). Results of the present study reveal that EIH occurs even after 45 min particularly after the highly intensive protocols (i.e. 100 and 110%). Hence, the intensity might also be a decisive variable that mediates the duration of EIH. Yet, concluding from these conflicting results, future studies should also implement pain sensitivity measurements not only immediately after the exercise, but also somewhat afterwards, e.g. 15, 30, 45, and 60-min post-exercise.

The present study further evaluated the effect of the different exercise session on CPM to investigate the endogenous pain-inhibitory system by determining the suppressive influence of a painful conditioning stimulus on a pre-applied painful test stimulus. Results indicate that none of the exercise sessions induced any change in CPM, leading to the assumption that endogenous inhibitory processes were only active to a limited degree to explain the hypoalgesia observed. This in line with existing literature stating that CPM cannot be seen as a primary mechanism of EIH (Ellingson et al. 2014). However, some influencing factors need to be discussed in this context. First, the exercises led to an increase in body temperature, which might have blurred the CPM results as for the test and conditioned stimuli thermal stimuli were employed. Second, sensitivity of CPM might be limited to yield statistically significant differences within the recruited subject sample of this study as healthy and young subjects often reach maximum values (Hackett et al. 2020), which is also observed in our study.

### Strengths and limitations

The major strength of the present study is that four different exercise intensities were evaluated with respect to the EIH phenomenon in a relevant large number of participants. Yet, there are also some limitations that need to be discussed. First, we only included healthy young male participants to homogenize the participant sample (Petrini et al. 2015) and results are therefore not directly transferable to older subjects and women and especially not to any patient populations. In this context, no exclusion criteria were defined regarding the subjects’ aerobic capacity. Hence, a certain degree of heterogeneity with respect to the aerobic training status needs to be considered. No information was collected regarding other influencing environmental and psychological factors, e.g. sleep deprivation (Schrimpf et al. 2015). Though all experimental pain measurements were conducted by the same investigator, no blinding was performed. We further measured PPT at joint landmarks and comparisons with other studies are difficult to provide, as most studies measured PPT at musculature landmarks. However, at the same time, the body of literature regarding EIH is thus extended.

## Conclusion

This study for the first time investigated the effects of a 30-min bicycle ergometer exercise comparing four different exercise intensities (60, 80, 10, and 110% of the IAT) on EIH. A main time effect was observed for PPT measured at the elbow, knee, and ankle joints, as well as the sternum with higher values observed 5 min and 45 min post-exercise, respectively. This observation might be explained by local pain-inhibiting pathways and to limited degree by central mechanisms as no such effect was observed for the forehead. However, no interaction effects were observed indicating that no intensity-specific “dose–response effects” with respect to EIH occurred in this study. Besides, none of the exercises altered CPM indicating that the central endogenous pain-inhibitory system does not reveal any acute exercise effects for any intensity used.Author: Please check the edit to the sentence ‚ A main time effect was observed for PPT measured at both elbow,…’This sentence was adjusted. As "Conflict of interest" statement is mandatory for this journal, please provide the same.Dear Sir or Madam, tank you for this advice. Please add the following statement: "Conflict of interest: The authors declare no potential conflicts of interest with respect to the research, authorship, and/or publication of this article."

## Supplementary Information

Below is the link to the electronic supplementary material.

Supplementary file1 (DOCX 17 KB)

Supplementary file2 (DOCX 23 KB)

## Abbreviations

ANOVA: Analysis of variance
EIH: Exercise-induced hypoalgesia
η^2^_partial_: Partial eta squared
CPM: Conditioned pain modulation
GABA: Gamma-aminobutyric acid
IAT: Individual anaerobic threshold
NRS: Numerical rating scale
PAG: Periaqueductal grey
PPT: Pressure pain threshold
VO_2_max: Maximum rate of oxygen consumption
VO_2_peak: Peak rate of oxygen consumption
