# Supplementary material for: Exercise-induced hypoalgesia (EIH) in response to different exercise intensities
Source: Eur J Appl Physiol. 2022 Jul 9;122(10):2213–22. doi: 10.1007/s00421-022-04997-1 (PMC9463310; doi:10.1007/s00421-022-04997-1)
Supplement: Supplementary file 1 — Supplementary file1 (DOCX 17 KB) [file 421_2022_4997_MOESM1_ESM.docx]

Supplementary Material 1: p-values resulting from post hoc tests for pressure pain thresholds (PPT) at landmarks, where a main effect for the factor ‘time point’ was observed, measured in response to the four different exercise intensities within the respective landmark. IAT=Individuals’ anaerobic threshold

| Protocol | Landmark | p-value of post hoc (pre-post 5‘) | p-value of post hoc (pre-post 45‘) | p-value of post hoc (post 5‘ – post 45‘) |
| --- | --- | --- | --- | --- |
| 60% IAT | Sternum | 0.011 | 0.011 | ns |
|  | Elbow left | ns | ns | ns |
|  | Elbow right | ns | ns | ns |
|  | Knee left | ns | ns | ns |
|  | Knee right | ns | ns | ns |
|  | Ankle left | 0.038 | ns | ns |
|  | Ankle right | 0.044 | ns | ns |
|  |  |  |  |  |
| 80% IAT | Sternum | ns | ns | ns |
|  | Elbow left | ns | ns | ns |
|  | Elbow right | ns | ns | ns |
|  | Knee left | ns | ns | ns |
|  | Knee right | ns | ns | ns |
|  | Ankle left | <0.001 | <0.001 | ns |
|  | Ankle right | 0.003 | <0.001 | ns |
|  |  |  |  |  |
| 100% IAT | Sternum | ns | ns | ns |
|  | Elbow left | 0.002 | 0.001 | ns |
|  | Elbow right | 0.004 | <0.001 | ns |
|  | Knee left | 0.042 | ns | ns |
|  | Knee right | ns | ns | ns |
|  | Ankle left | 0.026 | 0.015 | ns |
|  | Ankle right | 0.029 | 0.001 | ns |
|  |  |  |  |  |
| 110% IAT | Sternum | 0.007 | 0.027 | ns |
|  | Elbow left | 0.028 | 0.005 | ns |
|  | Elbow right | ns | 0.040 | ns |
|  | Knee left | 0.004 | ns | ns |
|  | Knee right | 0.001 | 0.008 | ns |
|  | Ankle left | 0.001 | 0.002 | ns |
|  | Ankle right | 0.002 | 0.040 | ns |
