# Supplementary material for: Exercise-induced hypoalgesia (EIH) in response to different exercise intensities
Source: Eur J Appl Physiol. 2022 Jul 9;122(10):2213–22. doi: 10.1007/s00421-022-04997-1 (PMC9463310; doi:10.1007/s00421-022-04997-1)
Supplement: Supplementary file 2 — Supplementary file2 (DOCX 23 KB) [file 421_2022_4997_MOESM2_ESM.docx]

Supplementary Material 2: Coefficients of variation resulting from PPT measurements presented for the four exercise protocols.

|  |  | 60% IAT | | | | 80% IAT | | | | 100 %IAT | | | | 110 % IAT | | | |
| --- | --- | --- | --- | --- | --- | --- | --- | --- | --- | --- | --- | --- | --- | --- | --- | --- | --- |
|  |  |  |  |  |  |  |  |  |  |  |  |  |  |  |  |  |  |
|  |  | **Min** | **Max** | **Mean** | **Standard deviation** | **Min** | **Max** | **Mean** | **Standard deviation** | **Min** | **Max** | **Mean** | **Standard deviation** | **Min** | **Max** | **Mean** | **Standard deviation** |
| pre | forehead | 0.01 | 0.29 | 0.07 | 0.07 | 0.01 | 0.27 | 0.07 | 0.05 | 0.02 | 0.27 | 0.08 | 0.05 | 0.01 | 0.58 | 0.08 | 0.11 |
|  | sternum | 0.01 | 0.29 | 0.07 | 0.06 | 0.01 | 0.52 | 0.08 | 0.10 | 0.00 | 0.35 | 0.06 | 0.07 | 0.01 | 0.48 | 0.08 | 0.10 |
|  | left elbow | 0.00 | 0.16 | 0.04 | 0.04 | 0.00 | 0.14 | 0.04 | 0.03 | 0.00 | 0.13 | 0.04 | 0.03 | 0.00 | 0.27 | 0.06 | 0.06 |
|  | right elbow | 0.00 | 0.18 | 0.05 | 0.04 | 0.00 | 0.17 | 0.04 | 0.04 | 0.00 | 0.10 | 0.04 | 0.03 | 0.00 | 0.32 | 0.05 | 0.06 |
|  | left knee | 0.00 | 0.29 | 0.06 | 0.07 | 0.00 | 0.08 | 0.03 | 0.02 | 0.00 | 0.17 | 0.05 | 0.04 | 0.00 | 0.21 | 0.06 | 0.06 |
|  | right knee | 0.00 | 0.22 | 0.05 | 0.05 | 0.00 | 0.16 | 0.04 | 0.04 | 0.00 | 0.20 | 0.05 | 0.05 | 0.00 | 0.21 | 0.05 | 0.05 |
|  | left ankle | 0.01 | 0.23 | 0.06 | 0.06 | 0.01 | 0.18 | 0.05 | 0.04 | 0.01 | 0.11 | 0.05 | 0.03 | 0.00 | 0.14 | 0.05 | 0.04 |
|  | right ankle | 0.00 | 0.16 | 0.05 | 0.04 | 0.01 | 0.10 | 0.05 | 0.03 | 0.00 | 0.30 | 0.06 | 0.06 | 0.01 | 0.16 | 0.04 | 0.03 |
| post 5' | forehead | 0.02 | 0.16 | 0.07 | 0.04 | 0.02 | 0.17 | 0.07 | 0.04 | 0.01 | 0.12 | 0.06 | 0.04 | 0.01 | 0.24 | 0.06 | 0.05 |
|  | sternum | 0.01 | 0.23 | 0.08 | 0.07 | 0.01 | 0.27 | 0.07 | 0.05 | 0.01 | 0.14 | 0.07 | 0.04 | 0.01 | 0.34 | 0.07 | 0.07 |
|  | left elbow | 0.00 | 0.15 | 0.04 | 0.04 | 0.00 | 0.19 | 0.05 | 0.04 | 0.00 | 0.17 | 0.04 | 0.04 | 0.00 | 0.21 | 0.05 | 0.06 |
|  | right elbow | 0.00 | 0.13 | 0.04 | 0.04 | 0.00 | 0.21 | 0.05 | 0.05 | 0.00 | 0.14 | 0.05 | 0.04 | 0.00 | 0.30 | 0.05 | 0.06 |
|  | left knee | 0.00 | 0.11 | 0.03 | 0.02 | 0.00 | 0.11 | 0.04 | 0.02 | 0.00 | 0.11 | 0.04 | 0.03 | 0.00 | 0.13 | 0.03 | 0.04 |
|  | right knee | 0.00 | 0.16 | 0.04 | 0.04 | 0.00 | 0.09 | 0.03 | 0.03 | 0.00 | 0.16 | 0.04 | 0.04 | 0.00 | 0.23 | 0.04 | 0.06 |
|  | left ankle | 0.00 | 0.13 | 0.05 | 0.03 | 0.00 | 0.14 | 0.04 | 0.03 | 0.00 | 0.22 | 0.05 | 0.05 | 0.00 | 0.21 | 0.04 | 0.04 |
|  | right ankle | 0.00 | 0.11 | 0.05 | 0.03 | 0.00 | 0.13 | 0.05 | 0.03 | 0.00 | 0.24 | 0.06 | 0.05 | 0.00 | 0.28 | 0.05 | 0.06 |
| post 45' | forehead | 0.00 | 0.25 | 0.07 | 0.05 | 0.01 | 0.17 | 0.06 | 0.04 | 0.01 | 0.15 | 0.06 | 0.03 | 0.01 | 0.24 | 0.06 | 0.05 |
|  | sternum | 0.01 | 0.15 | 0.05 | 0.03 | 0.01 | 0.16 | 0.05 | 0.04 | 0.00 | 0.16 | 0.06 | 0.03 | 0.01 | 0.17 | 0.05 | 0.03 |
|  | left elbow | 0.00 | 0.08 | 0.03 | 0.03 | 0.00 | 0.18 | 0.04 | 0.04 | 0.00 | 0.19 | 0.06 | 0.05 | 0.00 | 0.13 | 0.04 | 0.03 |
|  | right elbow | 0.00 | 0.14 | 0.03 | 0.03 | 0.00 | 0.12 | 0.05 | 0.03 | 0.00 | 0.07 | 0.03 | 0.02 | 0.00 | 0.16 | 0.05 | 0.04 |
|  | left knee | 0.00 | 0.22 | 0.04 | 0.05 | 0.00 | 0.08 | 0.03 | 0.02 | 0.02 | 0.21 | 0.07 | 0.05 | 0.00 | 0.10 | 0.05 | 0.03 |
|  | right knee | 0.00 | 0.12 | 0.05 | 0.03 | 0.00 | 0.26 | 0.04 | 0.06 | 0.01 | 0.20 | 0.10 | 0.06 | 0.00 | 0.13 | 0.04 | 0.04 |
|  | left ankle | 0.01 | 0.23 | 0.05 | 0.05 | 0.00 | 0.12 | 0.05 | 0.03 | 0.00 | 0.17 | 0.04 | 0.04 | 0.01 | 0.15 | 0.05 | 0.04 |
|  | right ankle | 0.01 | 0.08 | 0.04 | 0.02 | 0.00 | 0.22 | 0.04 | 0.04 | 0.01 | 0.19 | 0.05 | 0.05 | 0.00 | 0.34 | 0.05 | 0.07 |
